# Conserved and diverged asymmetric gene expression in the brain of teleosts

**DOI:** 10.3389/fcell.2022.1005776

**Published:** 2022-09-21

**Authors:** Carolina Agostini, Anja Bühler, Alessandra Antico Calderone, Narendar Aadepu, Cathrin Herder, Felix Loosli, Matthias Carl

**Affiliations:** ^1^ Department of Cellular, Computational and Integrative Biology (CIBIO), University of Trento, Trento, Italy; ^2^ Institute of Biological and Chemical Systems, Biological Information Processing (IBCS-BIP), Karlsruhe Institute of Technology, Karlsruhe, Germany; ^3^ Centre for Organismal Studies, Heidelberg University, Heidelberg, Germany

**Keywords:** brain asymmetry, medaka, zebrafish, habenula, pineal, parapineal, population genetics, kctd12.1

## Abstract

Morphological left-right brain asymmetries are universal phenomena in animals. These features have been studied for decades, but the functional relevance is often unclear. Studies from the zebrafish dorsal diencephalon on the genetics underlying the establishment and function of brain asymmetries have uncovered genes associated with the development of functional brain asymmetries. To gain further insights, comparative studies help to investigate the emergence of asymmetries and underlying genetics in connection to functional adaptation. Evolutionarily distant isogenic medaka inbred lines, that show divergence of complex traits such as morphology, physiology and behavior, are a valuable resource to investigate intra-species variations in a given trait of interest. For a detailed study of asymmetry in the medaka diencephalon we generated molecular probes of ten medaka genes that are expressed asymmetrically in the zebrafish habenulae and pineal complex. We find expression of eight genes in the corresponding brain areas of medaka with differences in the extent of left-right asymmetry compared to zebrafish. Our marker gene analysis of the diverged medaka inbred strains revealed marked inter-strain size differences of the respective expression domains in the parapineal and the habenulae, which we hypothesize may result from strain-specific gene loss. Thus, our analysis reveals both inter-species differences but also intra-species plasticity of gene expression in the teleost dorsal diencephalon. These findings are a starting point showing the potential to identify the genetics underlying the emergence and modulations of asymmetries. They are also the prerequisite to examine whether variance in habenular gene expression may cause variation of behavioral traits.

## Introduction

Brain asymmetries are present throughout bilateria. From insects to humans the morphologies of multiple parts of the central nervous system differ between the two sides of the body (Frasnelli et al., 2012; Rogers, 2014; Duboc et al., 2015; Alqadah et al., 2018; Kong et al., 2018). Given the widespread existence of asymmetries in the animal kingdom, it is likely that morphological and functional asymmetries are often associated and are advantageous for neuronal computation and the animals’ performance, well-being and behavior (Vallortigara and Rogers, 2005; Rogers, 2014; Güntürkün and Ocklenburg, 2017; Miletto Petrazzini et al., 2020). However, for the majority of brain asymmetries a functional relevance remains to be identified. For instance, numerous reports find links of asymmetry alterations to mental disorders, while others do not support this idea (Mundorf et al., 2021). Putative causative genetic mechanisms underlying such differences in left-right brain asymmetry have remained largely elusive. One approach to assess this enigma is to investigate the emergence of morphological brain asymmetries in connection to functional adaptation and the underlying genetics using comparative approaches. Teleosts show the highest radiation amongst vertebrates with diverged species that adapted to habitats with widely different conditions (Wittbrodt, Meyer and Schartl, 1998; Volff, 2005; Braasch et al., 2014). Importantly, a plethora of molecular genetic tools and resources are available to study phenotypic traits and molecular mechanisms in many teleost species.

Unlike numerous rather subtle left-right brain asymmetries, the dorsal diencephalon of teleosts is a remarkable exception as evidenced in numerous studies using the zebrafish (*Danio rerio*). The pineal complex consists of a centrally positioned pineal organ and a group of left-sided parapineal cells (Concha and Wilson, 2001; Beretta et al., 2012). In addition, also the adjacent bilaterally formed habenulae exhibit pronounced asymmetries. The neuronal subpopulations of the dorsal habenulae (dHb), which correspond to the mammalian medial habenulae (Amo et al., 2010), differ substantially between hemispheres with respect to size, gene expression, connectivity and function (Gamse et al., 2002; Concha et al., 2003; Gamse et al., 2003; Aizawa et al., 2005; Bianco et al., 2008; Miyasaka et al., 2009; Agetsuma et al., 2010; Dreosti et al., 2014; Krishnan et al., 2014; Facchin et al., 2015; Zhang et al., 2017). How these asymmetries have emerged and facilitate functional adaptation is largely unknown.

In view of the high divergence within the teleost clade a comparative study of diverged species will provide insights into the evolution of organs and underlying genetic mechanisms (Furutani-Seiki and Wittbrodt, 2004). The Japanese Killifish medaka (*Oryzias latipes*) is ideally suited for such studies due to its divergence from zebrafish of more than 100 Mya of independent evolution. Furthermore, medaka is a fully established laboratory teleost model with a long history of genetic studies (Takeda and Shimada, 2010). For example, several highly inbred isogenic strains, derived from different medaka wild populations, are available. A molecular genetic toolbox has been established that allows genetic studies, detailed analysis of gene function and genome editing (Kirchmaier et al., 2015). Most of the protocols established for zebrafish can be used for medaka with minor modifications. Thus, direct comparisons of reporter gene constructs are possible between these two teleost species.

Medaka also permits studies of intra-species variation, which is the basis of population genetic studies. The inbred strains from diverged medaka populations allow to study the plasticity of quantitative traits within this species. For example, using such inbred medaka strains, a genetic basis of strain-specific craniofacial variance has been shown (Kimura et al., 2007). Furthermore, a genetic contribution to the variation of brain morphology was reported in medaka (Ishikawa at al., 1999). Also, behavioral traits have been analysed by comparing isogenic inbred strains, suggesting that startle behavior in medaka is defined by intraspecific genetic polymorphisms (Tsuboko et al., 2014). Recently, a population genetic resource has been established, consisting of a panel of 80 inbred strains derived from the same wild population (Fitzgerald et al., 2022). Thus, medaka offers unique genetic resources to further our knowledge of intra-species variation in complex traits.

In this study, we used medaka as a diverged teleost for a comparative analysis of brain asymmetry. Using whole mount *in situ* hybridization (WISH) we examined the expression of a select number of marker genes that have been shown to be asymmetrically expressed in the developing diencephalon of zebrafish. We isolated and analysed 10 genes, 8 of which we find expressed in the medaka dorsal diencephalon. All of the commonly used markers for the pineal/parapineal complex in zebrafish, *otx5l, sox1a* and *gfi1ab,* are similarly expressed in medaka. Regarding the habenular specific genes, we observed largely conserved expression of the habenular precursor cell marker *cxcr4*, while the markers for habenular neuron subpopulations, *prox1a*, *cntn2, nrp1* and *kctd12.1,* differed in their extent of left-right asymmetry. We additionally revealed pronounced intraspecies variation of asymmetric gene expression by comparing two isogenic medaka strains from southern and northern populations, respectively, iCab and Kaga (Spivakov et al., 2014). In Kaga embryos, *otx5l* and *sox1a* expression in the parapineal are enlarged, while *kctd12.1* is much more prominently expressed, particularly in right dorsal habenular neurons compared to iCab embryos. These findings hint at intraspecific plasticity of left-right asymmetry.

## Materials and methods

### Fish maintenance and strains

The medaka inbred strains iCab and Kaga were kept in closed stocks at the Institute of Biological and Chemical Systems, Biological Information Processing (IBCS-BIP) at the Karlsruhe Institute of Technology (KIT), as previously described (Loosli et al., 2000). Medaka fish strains were maintained and bred according to standard procedures (Kinoshita et al., 2009). Medaka embryos were raised in a Petri dish containing ERM medium for 4 days at 28°C with a 14 h light/10 h dark cycle. Developmental stages were determined according to Iwamatsu, 2004.

### cDNA cloning

cDNA fragments of gfi1ab (Gene ID: 110015008), lov/kctd12.1 (Gene ID: 101161445), otx5l (Gene ID: 101164666), cntn2 (Gene ID: 101161878), prox1b (Gene ID: 100302040), cxcr4 (Gene ID: 100049444), nrp1 (Gene ID: 101161516), sox1a (Gene ID: 100304447), slc17a6b/vglut2 (Gene ID: 101175075), slc18a3b/vachtb (Gene ID: 101163117) were generated from cDNA libraries extracted from one to six dpf iCab embryos. Because zebrafish PROX1a and medaka PROX1b share the highest sequence homology with 71.80% and developmental gene expression, we renamed medaka prox1b to prox1a. The following primers were used: gfi1ab (Fw: 5′-GAG​TCG​GTG​GAT​AGG​TAC​GG; Rw: 3′-AGA​AGA​ATC​AGG​CTG​CCA​GT; 1185 bp cDNA), lov/kctd12.1 (Fw: 5′- CCG​CGT​TTC​CAT​CGA​ATC​AT; Rv: 3′-GCT​CAT​CTC​AGT​TCA​CAG​CA; 1155 bp cDNA), otx5l (Fw: 5′-GCA​CTC​CGA​GCA​GTT​TGA​AA; Rv: 3′-GAG​AGC​TGT​GAA​TGC​ATG​GG; 813 bp cDNA), cntn2 (Fw: 5′-GTC​ACG​GCC​ATG​ATA​ACC​AC; Rv: 3′-ACC​TGG​ATC​TCA​AAA​GCG​GA; 575 bp cDNA), prox1b (new prox1a) (Fw: 5′-AAC​CAG​CAT​GGG​TCA​GAG​AG; Rv: 3′-TTT​GAA​AGC​TGC​ACT​GAT​GG; 784 bp cDNA), cxcr4 (Fw: 5′-TTC​GAC​AAC​AGC​TCT​GAA​GG-3′; Rw: 3′-GGC​GTA​GAG​GAT​GGG​ATT​CA; 951 bp cDNA), nrp1 (Fw: 5′-TCA​TCT​CCT​ACA​CGG​GCA​TC; Rv: 3′-TCC​AAC​ACC​ATC​CTC​CAG​TC; 886 bp cDNA), sox1a (Fw: 5′-GAT​GAT​GGA​AAC​GGA​CCT​CC; Rv: 3′-AAA​TGT​GCG​TTA​GAG​GGA​CC; 956 bp cDNA), slc17a6b/vglut2 (Fw: 5′-TGT​GGG​GAT​GAT​TCA​TGG​CT; Rv: 3′-GAT​AGT​CCG​CTA​ACT​GGC​CT; 749 bp cDNA), slc18a3b/vachtb (Fw: 5′-TTG​TTT​GCG​TTG​CTC​TCC​TC; Rv: 3′-ACC​CAA​GGC​TCC​ATA​GAA​CC; 896 bp cDNA). For gfi, lov/kctd12.1, otx5l, cntn2 and prox1a cDNAs the following PCR program was used: 95°C for 5 min, 35 cycles of 95°C for 45 s, 58°C for 45 s, 72°C for 45 s and a final step at 72°C for 5 min. Cloning was performed with pCR™ 2.1-TOPO TA dual promoter cloning Kit (Invitrogen), following the manufacturer’s instructions. For cxcr4, nrp1, sox1a, slc17a6b/vglut2, slc18a3b/vachtb cDNAs the PCR program was the following: 98°C for 30 s, 35 cycles of 98°C for 30 s, 56°C for 30 s, 72°C for 30 s and the final step at 72°C for 30 s. Cloning was performed with Zero Blunt™ TOPO™ PCR cloning Kit (ThermoFisher), following the manufacturer’s instructions.

### Whole-mount *in situ* hybridization and antibody labeling

Whole-mount *in situ* hybridization has been performed using digoxigenin-labeled antisense RNA probes following standard procedures as described in Loosli et al. (1998) with the following modification: the temperature of hybridization and washing steps was 65°C.

For anti-acetylated tubulin immunostaining, four dpf embryos were fixed overnight at room temperature in 4% PFA/2x PBST and stored at −20°C in methanol. Samples were digested with a 10 μg/ml Proteinase K (Sigma-Aldrich) treatment for 45 min, refixed in 4% PFA/2x PBST, and blocked in PBS with 0.1% Tween20 and 5% normal goat serum (NGS). For antibody labeling, primary mouse anti-acetylated tubulin (1:1000, Sigma, #T6793) and secondary goat anti-mouse Alexa Fluor 488-conjugated (1:250, Invitrogen,# A11001) antibodies were used. For nuclear staining, embryos were incubated in PBS, 0.1% Tween20 containing DAPI (1:5000, ThermoFisher Scientific).

### Embryo pigmentation treatments

Pigmentation was prevented by treating growing embryos with 0.003% PTU/ddH_2_O (500 μl PTU/ddH_2_O solution in about 10 ml medium), added at 24 hpf in a Petri dish with 20–30 eggs per dish. The medium and the PTU were changed every day. Before immunolabeling, pigments were removed by incubating fixed dechorionated Kaga embryos at four dpf in freshly prepared 30% H_2_O_2_ and 1% KOH solution for up to several hours at RT.

### Microscopy and image manipulation

All *in situ* hybridization images were acquired by light microscopy using Zeiss Axio Imager M2 microscope and Keyence BZ-X810 microscope with a ×20 objective lens. Prior to acquisition, stained embryos were mounted in glycerol (Sigma-Aldrich) on microscope glass slides (Thermo Scientific).

For fluorescence confocal microscopy, embryos were mounted in 1% low-melt agarose in glass-bottom dishes (LabTek). Embryos were imaged using a TCS SP8 (Leica) inverse laser scanning microscope. Images were acquired with a ×20 air objective. Stack analysis, maximum intensity projections (MIPs), cropping and 2D-views were performed using Fiji und CS5 photoshop (Adobe) software.

### Area measurements and statistical analysis

Pineal complex, pineal and parapineal area measurements were performed with ImageJ-Fiji software using the Analyze Particles command. Habenula size was measured with ImageJ-Fiji by selecting and measuring the area of the habenula on confocal maximum projections stained with DAPI. The mean and standard error of the mean (SEM) were computed and plotted in a bar graph. The statistical analysis of the measured data was performed with the GraphPad Prism nine software, by applying the Mann–Whitney *U* test. Statistical values of *p*-value < 0.05 were considered significant.

## Results

### Expression of asymmetric habenular marker genes in medaka

**FIGURE 1:**
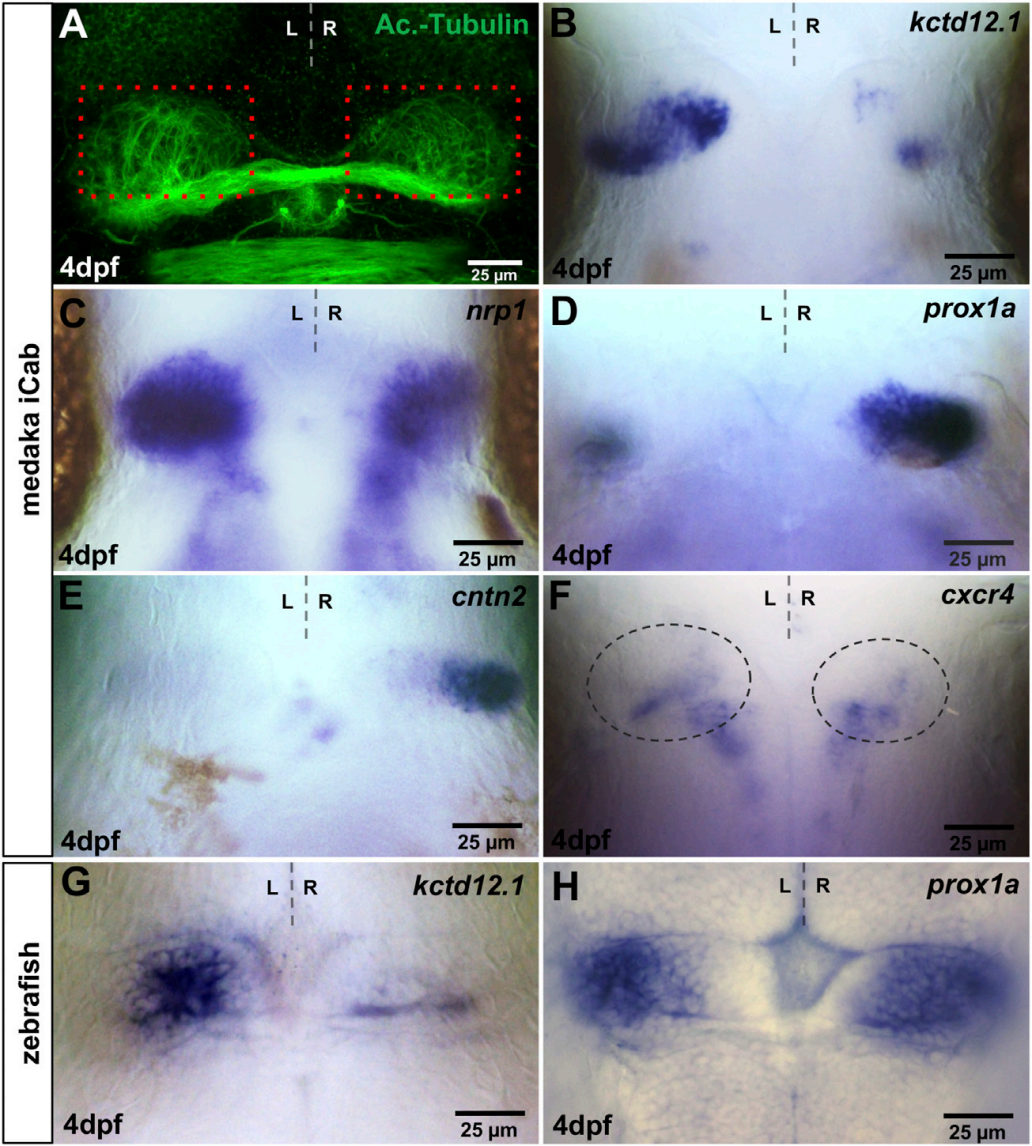
Visualisation of asymmetries in the medaka habenulae. iCab embryos at four dpf focused on the dorsal diencephalon; anterior to the top. **(A)** Immunostaining for anti-acetylated tubulin shows subtle left-right asymmetric dendritic arborisation in the habenula. Please compare the green signal within the same-sized red boxes. **(B–H)** Expression of habenula and pineal complex marker genes. **(B,C)** *kctd12.1* and *nrp1* are stronger expressed in left-sided habenular neurons. **(D,E)** *prox1a* and *cntn2* are mainly expressed in right-sided habenular neurons, while **(F)** *cxcr4b* is expressed in habenular progenitors, the area of which is encircled. **(G)** In the zebrafish, *kctd12.1* is expressed in lateral neurons of the dorsal habenulae, while **(H)** *prox1a* is expressed in medial dorsal habenular neurons.

Two main classes of morphological asymmetries can be distinguished (Concha et al., 2012): in class I, asymmetric structures differ in size between the two sides, while class II asymmetries comprise structures present only on one side of the body. The habenulae and the pineal complex exhibit class I and class II asymmetries respectively, the former of which can readily be visualized by immunolabeling of dendritic arborisation using anti-Acetylated Tubulin (Signore et al., 2009). We corroborated this finding by showing subtle, but reproducible, left-right differences in the extent of Acetylated Tubulin expressing habenular structures (Figure 1A). In zebrafish, numerous genes have been described that are either expressed specifically or largely specifically in subnuclei of the dorsal habenulae (dHb), in all dHb neurons or in dHb neuronal precursor cells. Of these, we isolated the medaka orthologues for *kctd12.1* (Gamse et al., 2003; Sato et al., 2021) and *nrp1* expressed predominantly in lateral neurons of the dorsal habenulae (dHbl) (Kuan et al., 2007), *cntn2* (Carl et al., 2007) and *slc18a3b/vachtb* as markers for medial neurons of the dorsal habenulae (dHbm) (Hong et al., 2013), *slc17a6b/vglut2* expressed in all habenular neurons (Thisse et al., 2005) and *cxcr4b* in dorsal habenular precursors (Roussigné et al., 2009). In addition, we re-assessed the expression of medaka *prox1a* (formerly named *prox1b*), which was reported to be right-dominantly asymmetric in the habenulae (Deguchi et al., 2009).

In four dpf old medaka iCab embryos, *kctd12.1* is expressed predominantly in left-sided habenular neurons, which likely correspond to the dHbl subnucleus (Figure 1B). The expression is enriched in a proximal and a distal cluster connected by cells with lower expression. On the right side, *kctd12.1* is weakly expressed in two small domains in a proximo-anterior and a distal-posterior cluster of cells differently from zebrafish, in which the gene exhibits a rather weak but continuous expression (Figure 1G) (Gamse et al., 2003). Also, medaka *nrp1* exhibits strong asymmetric left-sided expression in the dHb (Figure 1C). However, numerous dHb neurons express *nrp1* also on the right side, unlike its zebrafish orthologue (Kuan et al., 2007). In contrast to the left-sided habenular marker genes, we find strong *prox1a* expression in right habenular neurons as described (Figure 1D) (Deguchi et al., 2009), which likely represent dHbm neurons, whereas only a few cells on the left express *prox1a*. We re-assessed zebrafish *prox1a* expression (Thisse et al., 2005) and observed not only right-dominant expression of this gene’s mRNA in the habenula but also a prominent expression domain of *prox1a* on the left side (Figure 1H). Also, *cntn2* is expressed almost exclusively on the right side in four dpf iCab (Figure 1E), while in the zebrafish habenulae it is also expressed, albeit less prominently, on the left side (Carl et al., 2007). Therefore, our two chosen dHbm marker genes are more restricted to right-sided neurons in medaka compared to zebrafish. *cxcr4b* expression in habenular precursors in zebrafish (Roussigné et al., 2009) is downregulated during habenular maturation. Similarly, we find only a few cells expressing this gene in the iCab habenula at four dpf (Figure 1F). Our *slc18a3b/vachtb* probe did not detect any expression, while we found *slc17a6b/vglut2* expression in various brain regions at four dpf, in particular in telencephalic structures, but not in the habenulae (Supplementary Figure S1).

### Expression of pineal complex genes in medaka

**FIGURE 2:**
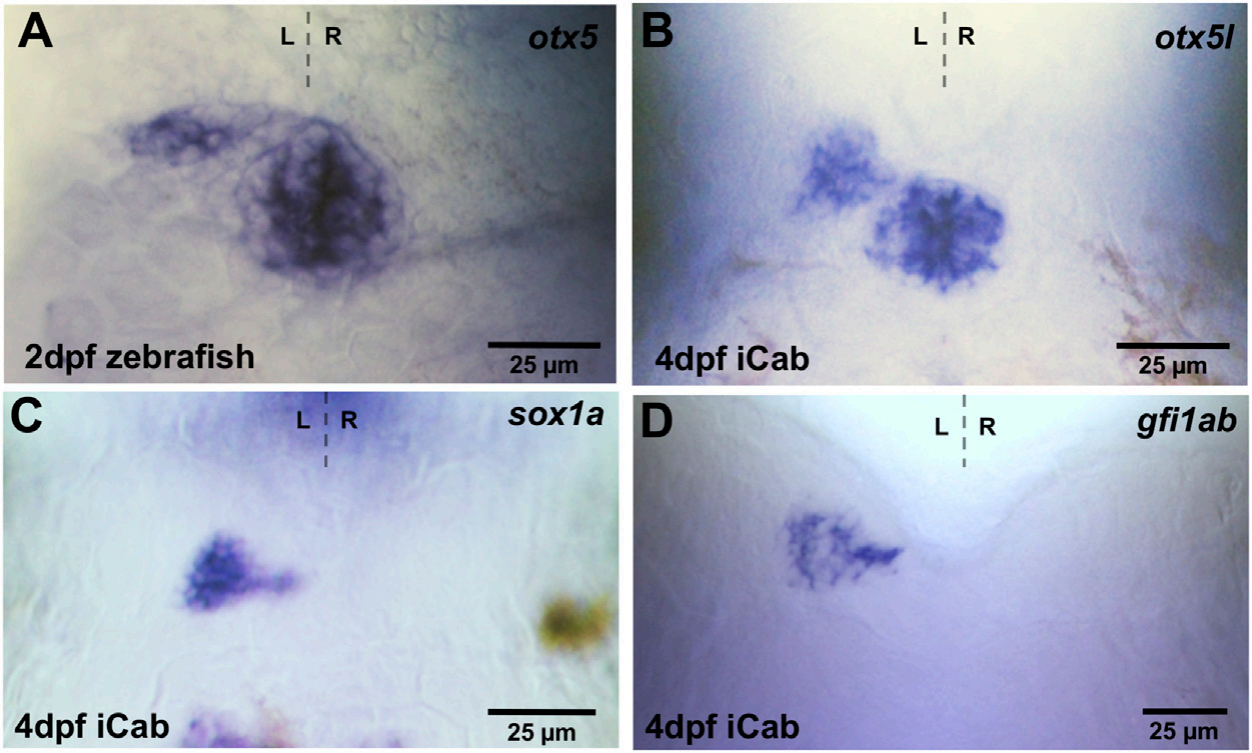
Expression of pineal and parapineal marker genes in the pineal complex. Zebrafish and iCab embryos at stages indicated focused on the dorsal diencephalon; anterior to the top. **(A,B)** *otx5*/*otx5l* is expressed in the pineal and parapineal in zebrafish and medaka, while **(C,D)** *sox1a* and *gfi1ab* are specifically expressed in the parapineal.

Zebrafish *otx5* is expressed in both pineal and parapineal cells (Figure 2A) (Gamse et al., 2002), while *sox1a* (Clanton et al., 2013) and *gfi1* (*gfi1ab*) (Dufourcq et al., 2004) are expressed exclusively in zebrafish parapineal cells. We isolated the respective medaka orthologs and found conserved *otx5l* transcripts in both pineal and left-sided parapineal cells of iCab embryos at four dpf (Figure 2B). As described previously, analyzing the expression of a Rx2:GFP transgene (Signore et al., 2009), the medaka parapineal volume judged by *otx5l* labeling appears to be slightly larger than the zebrafish parapineal. In analogy to zebrafish, *sox1a* and *gfi1ab* transcripts were detected specifically in medaka parapineal cells including a few cells seemingly connecting the parapineal with the pineal (Figures 2C,D). Therefore, the specificity of pineal complex genes is evolutionarily conserved between zebrafish and medaka.

### Northern and southern medaka inbred strains exhibit conserved and diverged gene expression patterns

**FIGURE 3:**
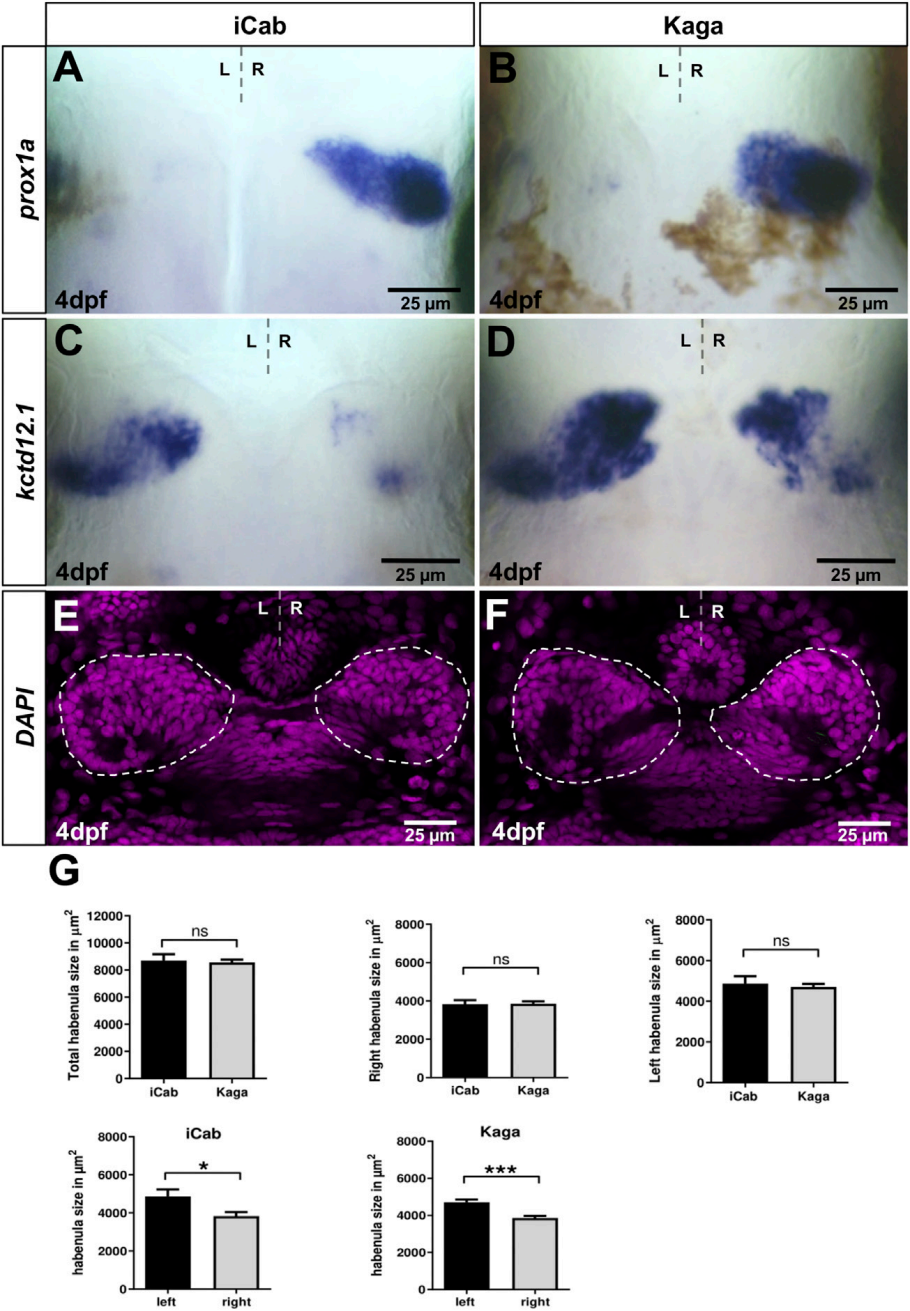
Habenular marker genes have conserved and diverged expression patterns in distant medaka strains. Dorsal views focused on the diencephalon at four dpf, anterior to the top. **(A,B)** *prox1a* expression in right-sided habenular neurons is comparable in iCab and Kaga, whereas **(C,D)** *kctd12.1* expression is particularly enlarged in right-sided habenular neurons in Kaga. **(E,F)** Single plane confocal images of the diencephalon of DAPI labeled embryos (n_iCab_ = 6; n_Kaga_ = 9). **(G)** No difference in habenular size between iCab and Kaga embryos, but a significant difference between the left and right habenulae in both populations was found. Dashed lines indicate the habenulae in **(E,F)**. Bar graphs show the means of the habenular areas. Error bars represent SEM (standard error of the mean).

Northern and southern medaka populations are diverged by at least 4 million years and show differences in various traits such as morphology and behavior (Takeda and Shimada, 2010; Tsuboko et al., 2014). In particular, the latter observation suggests that neural circuits are differently wired in these populations and cause such differences. Therefore, we assessed whether variations in gene expression are detectable in the dorsal diencephalic asymmetric neural circuit. Like observed in iCab embryos, we did not detect specific *slc18a3b* expression in Kaga embryos, while Please change into *slc17a6b/vglut2* is predominantly expressed in the telencephalon and not in the habenulae (data not shown). We find that *prox1a* expression in dHbm neurons is largely conserved between the southern iCab strain and the northern Kaga strain (Figures 3A,B). In contrast, *kctd12.1* is expressed in a larger domain on the left in Kaga embryos when compared to iCab (Figures 3C,D). In addition, the anterior-proximal expression domain on the right hemisphere is expanded in Kaga embryos. We verified that the differences in gene expression are not due to differences in habenular sizes between the two strains by measuring the habenular size by nuclear staining in embryos at four dpf (Figures 3E–G). We did not find any significant differences in the left, right or total habenular volume between iCab and Kaga. However, we noticed that in both strains the left habenula is about 20% larger than the right. This corresponds to previous estimates from marker gene expression and neuropil labelings in zebrafish (Concha et al., 2000; Gamse et al., 2003). Our habenular size measurement shows that the observed differences of *kctd12.1* expression between iCab and Kaga are not due to differences in habenular size.

**FIGURE 4:**
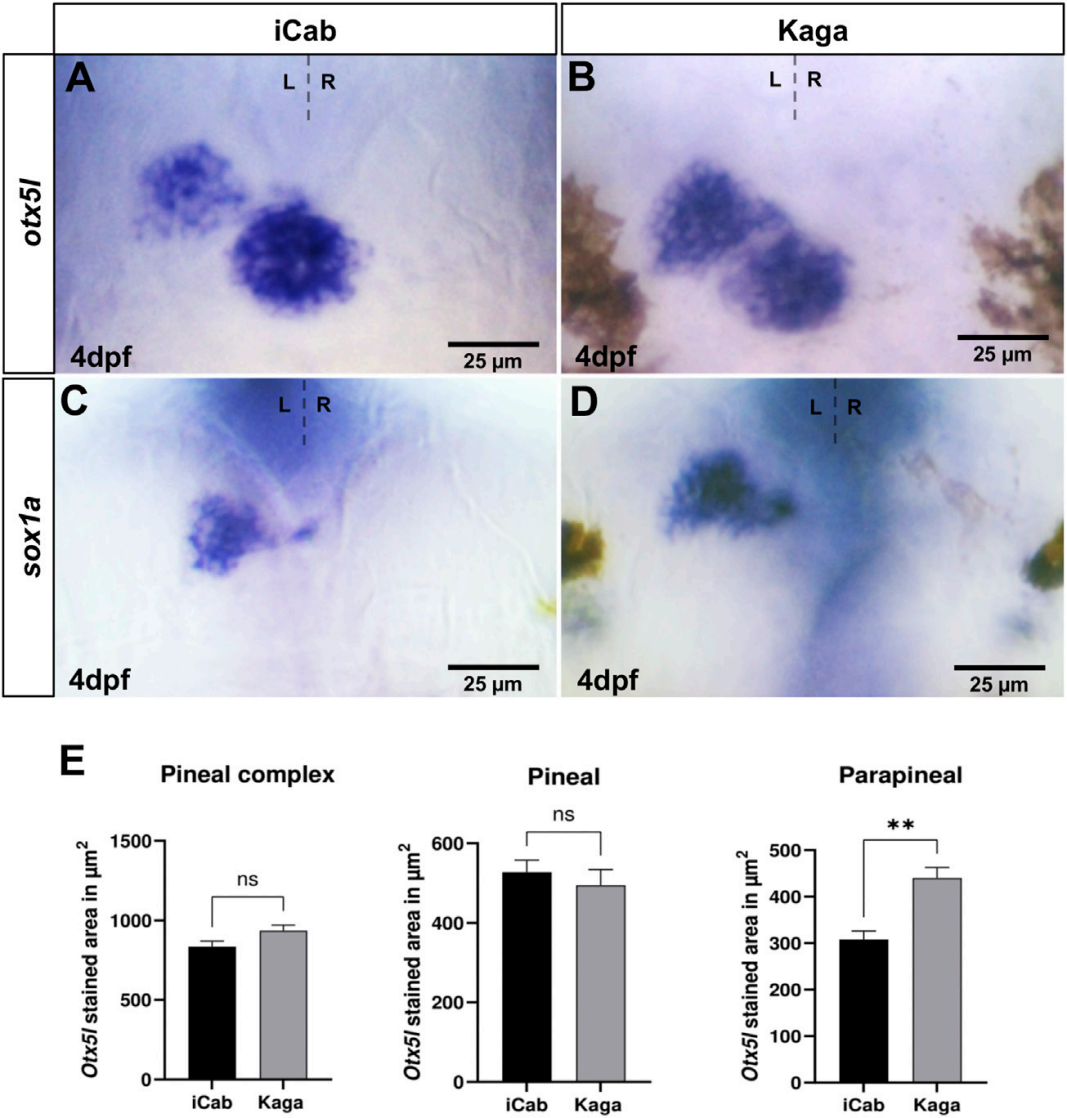
Parapineal size differs significantly between Kaga and iCab embryos. Dorsal views focused on the diencephalon of four dpf embryos with anterior to the top. **(A–D)** *otx5l* and *sox1a* are expressed in pineal/parapineal of both iCab and Kaga embryos. **(A,B,E)** The parapineal expression domain of *otx5l* is significantly larger in Kaga embryos compared to iCab embryos, whereas no size difference of the *otx5l* expression domain in the pineal alone or in the pineal/parapineal complex together was detected (n_iCab_ = 5; n_Kaga_ = 5). Bar graphs show the means of the pineal complex, pineal and parapineal areas. Error bars represent SEM (standard error of the mean).

Next, we compared gene expression in the pineal complex between the two medaka strains (Figures 4A,B). While the *otx5l* expression domain in the pineal alone or in the pineal/parapineal complex together does not significantly differ between iCab and Kaga, the parapineal expression domain is significantly larger in Kaga embryos (Figure 4E). Similarly, also the parapineal specific expression of *sox1a* is larger in Kaga embryos (Figures 4C,D). Taken together, our intra-species analysis of asymmetric diencephalic neuronal structures and associated gene expression reveals differences in the parapineal expression domains of *otx5l* and *sox1a* as well as of the dHbl marker *kctd12.1*, while dHbm marker gene expression appears conserved in embryos of northern and southern medaka populations.

## Discussion

Our study on marker gene expression in left-right asymmetric neuronal structures in the medaka diencephalon reveals conserved and species-specific differences in the teleost lineage. We find that five out of the seven genes, selected due to their specific expression in the zebrafish habenulae, are also expressed in the medaka habenulae. Notably, right dominant markers for dHbm neurons such as *prox1a* and *cntn2* are more restricted to this side in medaka iCab embryos than in zebrafish, while left-sided *nrp1* expression in dHbl neurons is more left-specific in zebrafish (Thisse et al., 2005; Carl et al., 2007; Kuan et al., 2007). Previous studies have identified roles for *cntn2* and *nrp1* in neuronal migration and axon fasciculation as well as axon guidance during zebrafish development, respectively (De Bruin et al., 2016; Gurung et al., 2018). Therefore, the findings of our comparative study could contribute to explain the rather subtle differences in the targeting of habenular efferent axons in the interpeduncular nucleus between medaka and zebrafish (Signore et al., 2009). Furthermore, all three pineal complex genes, *otx5l*, *sox1a* and *gfi1ab* show a largely conserved expression between medaka and zebrafish (Gamse et al., 2002; Dufourcq et al., 2004; Clanton et al., 2013).

Our comparison between diverged medaka inbred strains revealed different variations of gene expression in the asymmetric diencephalic structures investigated. Firstly, the parapineal, as defined by *otx5l* and *sox1a* expression, is bigger in the Kaga strain compared to iCab embryos. This is paralleled by a stronger expression of *kctd12.1* in left dHbl neurons and remarkably also on the right hemisphere of Kaga embryos. In contrast, *prox1a* expression in dHbm neurons exhibits the same pattern in Kaga and iCab habenulae suggesting that some right habenular neurons may express both genes in Kaga. This, in turn, might implicate different fates of habenular neurons on the right side between Kaga and iCab. Future connectome and functional analyses will be needed to test this hypothesis. Another question arising is whether the differences detected in parapineal size and dHbl marker gene expression between iCab and Kaga are separated evolutionary events or whether they are linked to one another and caused by a, perhaps more likely, single event. Many years of research in zebrafish have shown that dHbl neurogenesis and the parapineal are connected, although the precise mechanism is still not fully resolved, dHb neurons develop from multipotent pools of habenular precursor cells adjacent to the pineal complex. Influenced by temporally tightly regulated Wnt/beta-catenin signaling alongside Notch signaling, they develop into dHbm neurons (Aizawa et al., 2007; Carl et al., 2007; Beretta et al., 2013; Hüsken et al., 2014; Guglielmi et al., 2020). This predominantly happens in the right hemisphere. On the left half of the brain, parapineal cells migrate away from the pineal influenced by Nodal and Fgf signaling shortly before habenular precursors become post-mitotic and differentiate (Concha et al., 2003; Regan et al., 2009; Roussigné et al., 2018). It is thought that yet unknown signals released from these parapineal cells that depend on *sox1a* function (Lekk et al., 2019) influence habenular neuron precursors on the left side to adopt dHbl character (Concha et al., 2003; Hüsken et al., 2014). In this process, the size of the parapineal not only appears to influence left dHb precursors but, in certain circumstances, also the right habenula. *Pitx2* genes are members of the bicoid-class of paired homeodomain transcription factors and targets of Nodal signaling. In zebrafish, *pitx2c* is expressed in the left dorsal diencephalon and influences parapineal development (Essner et al., 2000; Garric et al., 2014). In *pitx2c* hypomorphic embryos, the parapineal cell number is increased. These embryos exhibit an enlarged domain of right-sided dHbl typical *kctd12.1* expression, while dHbm markers remain unaffected. This phenotype is remarkably similar to our observations in Kaga embryos and raises the hypothesis that evolutionary events may have caused modulations around the genomic interval comprising the Kaga *pitx2* gene. In medaka iCab embryos belonging to the southern population, the *pitx2* gene has been identified and its asymmetric expression in the brain described (Jaszczyszyn et al., 2007; Soukup et al., 2018). Regarding northern populations, only the medaka HNI genome sequence is currently available. Although validating our hypothesis certainly warrants the analysis of the Kaga genome, we performed a preliminary HNI genome analysis and could only detect high sequence homologies for *pitx1* and *pitx3* in the genome of zebrafish and medaka HNI. We did not detect any sequence homologies representing *pitx2* in the HNI genome (data not shown). Future functional experiments and the availability of a Kaga genome sequence will allow investigating whether the differences detected in parapineal size and *kctd12.1* gene expression in Kaga are linked and caused by a single event during evolution, which is the loss of *pitx2* in northern populations.

Our work has substantiated that medaka is particularly suitable for elucidating intra-species variations for population genetic studies. Here we used established inbred strains from diverged medaka populations to search for variance of the emergence of brain asymmetries. Based on our findings, we suggest that such intra-species comparisons can serve as a starting point to link brain asymmetries to adaptive behaviors in future studies. Behavioral tests in medaka to investigate anxiety-like behaviors and sociability have recently been reported (Lucon-Xiccato et al., 2022). Both of them are strongly influenced by habenular function and could therefore serve as a readout for habenular function (Bühler and Carl, 2021). In view of the recently established panel of inbred strains derived from a single wild population, medaka offers unique genetic resources to tackle this long-standing question in neuroscience (Fitzgerald et al., 2022).

## Data Availability

The original contributions presented in the study are included in the article/Supplementary Material, further inquiries can be directed to the corresponding authors.
