# Supplementary material for: Conserved and diverged asymmetric gene expression in the brain of teleosts
Source: Front Cell Dev Biol. 2022 Sep 21;10:1005776. doi: 10.3389/fcell.2022.1005776 (PMC9532764; doi:10.3389/fcell.2022.1005776)
Supplement: Supplementary file 1 [file Image1.pdf]

Supplementary Material

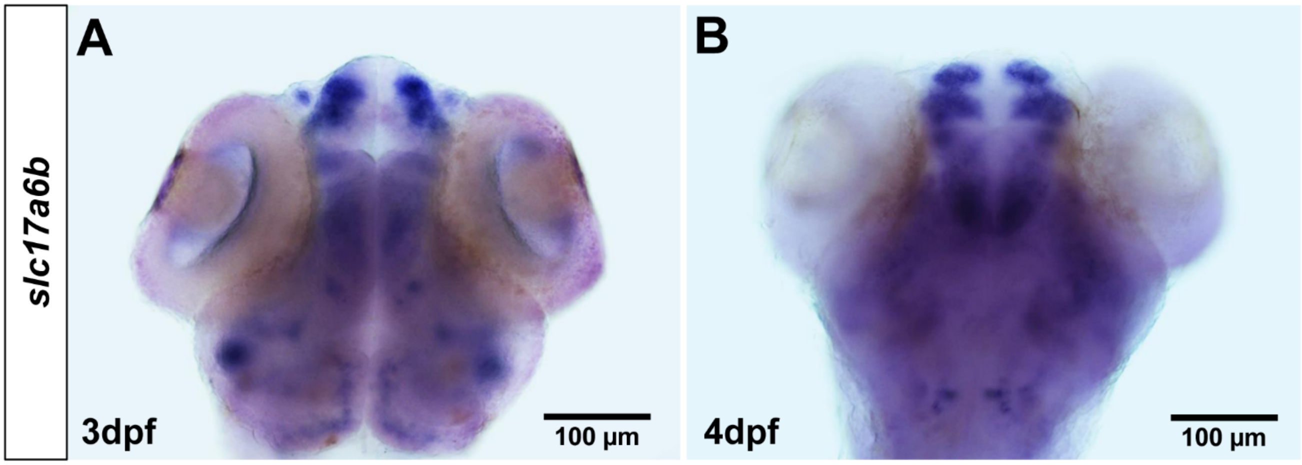

Supplementary Figure 1. *slc17a6b/vglut2* is not expressed in the medaka habenulae. (A, B) Dorsal views of 3 dpf and 4 dpf old iCab embryos focused on the head with anterior to the top. In the forebrain of iCab embryos, *slc17a6b* is mainly expressed in two distinct bilateral domains in the telencephalon.
